# The Influence of Intermittent Fasting on Selected Human Anthropometric Parameters

**DOI:** 10.7150/ijms.99116

**Published:** 2024-10-07

**Authors:** Erika Čermáková, Martin Forejt, Martin Čermák

**Affiliations:** 1 Department of Public Health, Faculty of Medicine, Masaryk University, Kamenice 753/5, 625 00 Brno, Czech Republic.; 2 Independent Researcher.

**Keywords:** intermittent fasting, time-restricted feeding, body fat mass, visceral fat, skeletal muscle mass, energy restriction

## Abstract

**Background:** Intermittent fasting may be an effective tool for weight loss, but it is still unclear from previous studies to date whether it is as effective as a continuous energy restriction in terms of reducing adipose tissue and whether it leads to unwanted muscle loss.

**Objectives:** The aim of this study was to compare the effect of intermittent fasting (IF) with continuous energy restriction (CER) on the body weight and body composition and to assess the effect of intermittent fasting also in isolation from the energy restriction.

**Methods:** After completion of a three-week dietary intervention, differences in the weight loss and differences in the body composition were compared between three groups. The first group consumed 75% of their calculated energy intake requirements in a six-hour time window. The second group consumed 75% of their calculated energy intake requirements without a time window and the third group consumed 100% of their calculated energy intake requirements in a six-hour time window. The changes in the weight and body composition were assessed by BIA.

**Results:** Of the 95 randomized participants, 75 completed the intervention phase of the study. The highest mean weight loss was achieved by the IF with ER (energy restriction) group (2.3 ± 1.4 kg), followed by the CER group (2.2 ± 1.1 kg); the difference between the groups did not reach statistical significance. The lowest mean weight loss was observed in the IF without ER group (1.1 ± 1.2 kg), the difference reaching statistical significance compared to the IF with ER (p=0.003) and CER (p=0.012) groups. The highest mean adipose tissue loss was observed in the CER group (1.5 ± 1.2 kg) followed by the IF with ER group (1.3 ± 1.1 kg), with no statistically significant differences between the groups. A mean adipose tissue loss was found in the IF without ER group (0.9 ± 1.1 kg) with no statistically significant differences compared to the IF with ER and CER groups. The highest mean fat-free mass loss was found in the IF with ER group (1.1 ± 1.0 kg), followed by the CER group (0.65 ± 0.91 kg) with no statistically significant differences. The IF without ER group showed the lowest mean fat-free mass loss (0.2 ± 1.3 kg), which reached statistical significance compared to the IF with ER group (p=0.027).

**Conclusion:** The results showed a comparable effect in the weight loss and body fat reduction regardless of the timing of the food intake. The diet quality, together with the energy intake, appeared to be one of the most important factors influencing the body composition.

## Introduction

The number of people who are overweight is constantly increasing, the obesity epidemic has become a global problem, led by the countries of the Western world 1. Therefore, numerous research studies have focused on how to effectively reduce one's body weight and improve the other parameters related to cardiometabolic health, such as the percentage of body fat and muscle mass, the amount of visceral fat, the waist circumference and the waist-to-hip ratio 2.

In simple terms, the cause of being overweight and obesity is the long-term predominance of energy intake over energy expenditure 3,4. The generally recommended method of reducing excess adipose tissue is continuous energy restriction, based on the regular consumption of three large main meals and two smaller snacks between the main meals per day. However, for many people trying to lose weight, this type of diet can be difficult to achieve. This may be due, for example, to the lack of time to prepare and eat regular meals due to the nature of work or shift work, an evening chronotype that makes it difficult to include breakfast, etc. Therefore, the research in recent years has focused on finding suitable alternatives to induce an energy deficit without undesirably eliminating essential nutrients or food groups. An increasingly popular alternative that has been described in many recent studies is intermittent fasting, where food is consumed only during a specific time window. This can lead to a reduction in the energy intake 5,6.

There are three basic types of intermittent fasting diets. The first two restrict the food intake on specific days of the week, either every other day (known as an alternate fasting day - AFD) or completely or significantly on two days of the week (known as 5:2). The third type of intermittent fasting reserves space for energy intake for a defined period of time each day, e.g., 4-8 hours for eating, 20-16 hours for fasting (called time-restricted feeding - TRF) 7.

Based on the results of many studies, we know that eating according to one of the above intermittent fasting protocols can be an effective tool for weight loss 8,9. However, the results are still unclear if intermittent fasting is more effective than continuous energy restriction without a time restriction, especially when it comes to reducing the body fat mass along while preserving the active muscle mass, which is important for sustaining results 10-12. This information is particularly important for assessing the suitability of such a diet as a sustainable lifestyle.

A number of studies have compared the effect of intermittent fasting with continuous energy restriction 13-17, other studies have attempted to isolate the effect of intermittent fasting from energy restriction by combining intermittent fasting with 100% energy intake without any energy restriction 10,18. Both of these studies were based on alternating a day of eating and a day of fasting. The authors of one of these studies concluded that intermittent fasting was less effective at reducing the fat mass than comparable continuous energy restriction 10. This way of eating can also lead to more significant adverse effects in the form of psychological and physical discomfort. For example, many people who fast all day may experience increased fatigue, difficulty concentrating, excessive hunger and cravings, etc. 12. To our knowledge, none of the studies have yet assessed, in isolation, the effects of fasting when food is consumed during a specific time window each day, which is closer in nature to the generally recommended principles of inducing an energy deficit and does not involve fasting for a long time period. For many individuals, this may be an easier and more sustainable option than significantly or completely restricting the food intake every other day or several days per week (AFD/2:5).

The aim of the study was to compare the effects of intermittent fasting with a defined period of food intake during the day with continuous energy restriction on the body weight and body composition, and to assess the effects of intermittent fasting in isolation from the energy restriction.

## Methods

### Participants

Healthy adult volunteers were included in the study. The inclusion criteria were over 18 years of age, interest, and willingness to spend time preparing and eating the meals according to the diet plan. The individuals whose condition or diagnosis could bias the results of the study or make participation in the study risky, or who could not consume the prescribed foods (vegans) were not included. The following exclusive criteria were defined: pregnancy and breastfeeding, pacemaker, insulin pump, underweight (BMI<18.5), patients with the following diagnoses: cancer, gout, grave gastrointestinal tract disease, kidney disease, diabetes mellitus, post-transplant patients, autoimmune disease, thyroid disease, eating disorders, and patients taking warfarin.

The study was conducted in accordance with the Declaration of Helsinki, approved by the Ethics Board of the Faculty of Medicine, Masaryk University (approval number 80/2022; date 25 November 2022). Informed consent was obtained from all the participants.

The participants were recruited via a media campaign. Radio and television media were contacted through a press release published on the faculty website and through the media's social networks. The campaign was aimed at the population of the Czech Republic. The recruitment of the participants was completed after successful completion of the intervention by the required number of volunteers in all the intervention groups.

*The sample size* was calculated based on information from a previous similar study 10, where the mean difference in the fat mass loss between two groups was 1 kg. The assumed standard deviation of the measurements was set at 1 kg (deviation at the level of the difference in the fat mass loss in the restriction group versus the control group). The probability of a false-positive result was assumed to be 5% and the power of the test was set at 80%. According to 19,20, the minimum sample size for the analysis of variance was calculated to be 16 individuals per group, for a total of at least 48 persons for all three groups. To enable comparison between groups using the Bonferroni test, a minimum of 21 participants per group was required, resulting in a total of 63 participants across the three comparison groups.

A total of 97 participants were included in the study, with 95 of them being randomly assigned to one of three intervention groups. In case that medical contraindications were identified during the initial interview, the participants were not further randomized (two individuals did not meet the inclusion criteria - gastrointestinal diseases). During the intervention phase, 20 participants withdrew from the study. Table 1 provides an overview of the reasons for the early withdrawal from the dietary intervention.

## Experimental design

### Initial consultation

All the volunteers were first briefed on all the details of the study before being included in the study. Informed consent was obtained from all the participants prior to their inclusion in the study. All the necessary data were collected during the interview and recorded on pre-prepared forms. Basic demographic information, health contraindications, food allergies, intolerances, and taste preferences were gathered. Additionally, the chronotype of the participants was investigated. All the subjects were assessed in detail for their physical activity, which included the most detailed information on their lifestyle and targeted physical activities.

### Anthropometric characteristics

Basic anthropometric methods were used to determine the current anthropometric characteristics. This included measuring their body height using a Seca 764 stadiometer (seca GmbH & Co., Hamburg, Germany) and determining their body composition through a bioelectrical impedance analysis (BIA) by an InBody 970 body composition device with the software Lookin´Body 3.0 (Biospace, Seoul, South Korea), which works on the principle of a direct segmental multifrequency BIA. The body composition analysis was conducted under standardized conditions to ensure the most accurate results 21. The body composition measurements were taken in underwear, including the removal of jewelry, watches, etc. The participants were instructed to abstain from food and drink for 2.5 hours prior to the measurement and to avoid engaging in high-intensity physical activity such as endurance running, swimming, or weight training. Before the measurements were taken, the subjects had an empty bladder. It was important to maintain the cleanliness of the hands and feet and avoid using greasy creams. The adherence to the above guidelines was confirmed prior to each measurement.

### Study design

The participants were randomly assigned to one of three intervention groups using block randomization, stratified by sex (female or male) and the body mass index (BMI) (<25; ≥25 or ≥30 kg/m^2^). The randomization scheme was prepared by a person who was not involved in the recruitment of the participants and who did not know their identification codes. According to the assigned group, each participant received an individual eating plan, which they followed for the next three weeks. The participants in the first intervention group consumed 75 % of their daily energy needs within a six-hour time window, the start of which was chosen by each participant (**IG1: TRF in combination with the ER, self-selected**). The second intervention group consumed 75 % of their energy needs without any time limitations (**IG2: CER**). The third intervention group consumed 100 % of their energy needs during a six-hour window, the start of which was chosen by each participant (**IG3: TRF alone, self-selected**). All three intervention groups required a minimum three-hour interval without food intake before going to sleep. The six-hour time window was selected to allow for two main meals per day, with the option of one snack between meals. The remaining 18 hours were spent fasting. This ensured that all the participants consumed all the necessary nutrients, while avoiding non-serious digestive problems such as bloating, heartburn, etc. after eating large amounts of food at once. Furthermore, each participant was given the option to select the beginning of their six-hour food intake window, which remained valid throughout the intervention period. This choice was based on their preference for a chronotype or usual daily routine. The duration of the intervention, three weeks, was designed to allow for the manifestation of the effect, while considering the time required to prepare the individual meals according to the diet plan of each participant.

### Diet plans

The diet plans were compiled by calculating the energy needs of each participant using NutriPro software (https://nutripro.cz/). In addition to the precise energy value, which was determined strictly on an individual basis, emphasis was also placed on ensuring sufficient protein intake at a rate of 1.2-1.6 g/kg of body weight 22, for participants with strength or very intensive sports training 3-4 hours per week, the protein intake was increased to 1.8-2 g/kg of body weight 23,24. Furthermore, all the diet plans contained a sufficient amount of dietary fiber at a minimum of 25-30 g/day 25,26. All the meal plans were designed with an emphasis on high quality meals that included representation from all the food groups per the food pyramid 27. The diet consisted of complex carbohydrate sources, including oatmeal, whole grain bread, rice, and pasta. In addition, sufficient fruit and vegetables were included in the minimum recommended amount of 500 g or more per day, with a predominance of vegetables. Dairy products were chosen as the protein sources and a source of calcium, with sour variants of dairy products and cheese being preferred. Other protein sources included fish, lean meat, eggs, and legumes. The main sources of added fat were nuts, rapeseed, and olive oil due to their high content of monounsaturated and polyunsaturated fatty acids. The meal plans were drawn up in three variants. This ensured a variety of food choices throughout the 21-day period and thus also ensured the adequate intake of micronutrients in the diet.

*The total daily energy expenditure of the participants* 28 (DEE - Daily energy expenditure) was determined by the basal metabolic rate (BMR), which was estimated from the body composition measurements using the BIA. Furthermore, the energy expenditure of the physical activity was added. The lifestyle activity (employment, housework, walking) and targeted physical activity (cycling, weight training, ball games, etc.) were included in the calculation. The average weekly physical activity level (PAL) was determined based on detailed data, following the recommendations of the European Food Safety Authority 29. The final component of the energy expenditure was the diet-induced thermogenesis (DIT) effect, which was determined to be 10 % on a mixed diet with sufficient protein 22,30. Based on the above parameters, the daily energy expenditure of the participants can be determined using the following equation:

DEE=BMR*(PAL_lifestyle_ + PAL_targeted physical activities_)*DIT

Based on the meal plan, all the information regarding the preparation and consumption of the meals was explained to the participants in detail. Particular foods were demonstrated in pictures using a food catalog. The participants were explained how to measure the weights of the different foods and how to prepare the dishes.

*The drinking regimen* of the participants during the time window for the food intake and outside the time window included only water or, alternatively, unsweetened tea and coffee. The participants were asked to monitor their fluid intake by the color of their urine (if the urine was too dark, they needed to drink). This not only ensured sufficient fluid intake, but that it was also evenly distributed throughout the day.

### Intervention phase

During the intervention phase of the study, the participants were contacted by phone every week (one week after the start of the intervention and then one week before the end of the intervention) to check on the progress of the intervention and to keep the participants motivated. Any relevant information found was recorded on pre-prepared forms. The focus was on ascertaining adherence to the established dietary regimen. The potential occurrence of adverse events related to the changes in the diet and nutrition was also monitored. If the participants failed to adhere to the prescribed fasting and food intake schedule, their participation in the study was terminated prematurely.

### Final outcomes

After the three-week dietary intervention, the participants were invited for final measurements to assess their current anthropometric characteristics. In addition to the measured changes in the body composition, the course of the dietary intervention in the third week was also monitored, including any adverse events. The information of the concluding interviews was documented on pre-prepared forms.

### Statistical methods

The statistical analysis of the results was performed using Statistica software (TIBCO® Statistica™ Version 14.0.0.15, Palo Alto, CA, USA). The statistically processed parameters are presented as the arithmetic mean ± standard deviation (SD) unless otherwise stated (see Tables 2 and 3). The statistical significance was set at p<0.05 for all the analyses. Levene's test was used to assess the homogeneity of variance of the data between the groups. The normality of the resulting differences in the participant's weight before and after the intervention was tested using the Lilliefors test. The Shapiro-Wilk test was used to test the normality of the other anthropometric data. An analysis of variance (ANOVA) was used to test the comparisons of the outcome data between the intervention groups. Three post-hoc tests were conducted with Bonferroni correction for pairwise comparisons among all the groups. The difference between the pre- and post-intervention values is denoted by a letter (“Δ”). For categorical variables, we tested the statistical significance using the chi-square test, specifically the Fisher exact test.

## Results

### Characteristics of the study participants

Out of the 95 participants who were randomized, 75 completed the intervention phase of the study. The characteristics of the study population are presented in Table 2. The mean age (± SD) of the participants was 37 ± 10 years, the mean weight was 83 ± 16 kg, the BMI was 28.4 ± 4.4, the BF% was 32.4 ± 8.3, the mean PAL was 1.63 ± 0.13, and the BMR was 1580 ± 260 kcal.

### Effect of the dietary interventions on the weight loss

The highest average weight loss was measured in the group of participants with intermittent fasting and energy restriction, IG1 (2.3 ± 1.4 kg), a comparable loss was observed in the group with continuous energy restriction, IG2 (2.2 ± 1.1 kg). The difference between those two groups did not reach statistical significance (p>0.99). The lowest mean weight loss was observed in the group of participants with intermittent fasting without energy restriction, IG3 (1.1 ± 1.2 kg). The results achieved statistically significant differences between the energy restriction with the time restriction group of participants (IG1×IG3: p=0.003) also without the time restriction (IG2×IG3: p=0.012) compared to the group without the energy restriction. Statistically significant differences were also observed when analyzing the measured changes in the BMI (p=0.008 for the difference IG2×IG3).

### Effect of the dietary interventions on the body composition

#### Body fat mass and body fat percentage

The greatest average loss of measured body fat mass compared to the baseline was found in the group of participants with the continuous energy restriction, IG2 (1.5 ± 1.2 kg), followed by the group with the intermittent fasting and energy restriction, IG1 (1.3 ± 1.1 kg). The lowest average body fat mass loss was found in the group of participants with the intermittent fasting and without the energy restriction, IG3 (0.9 ± 1.1 kg). However, the differences between the groups did not reach statistical significance in any of the three groups (IG1×IG2: p>0.99; IG1×IG3: p=0.73 a IG2×IG3: p=0.17). Comparable results were also found for the body fat percentage.

#### Visceral fat area

The greatest average loss of measured visceral fat compared to the baseline was found in the group of participants with the continuous energy restriction, IG2 (9.3 ± 6.6 cm^2^), followed by the group with the intermittent fasting and energy restriction, IG1 (6.8 ± 6.3 cm^2^). The lowest average visceral fat loss was found in the group of participants with the intermittent fasting and without the energy restriction, IG3 (4.6 ± 5.8 cm^2^). A statistically significant difference was achieved between the group of participants with the continuous energy restriction and the group with the intermittent fasting without the energy restriction (IG2×IG3: p=0.043). No statistically significant difference was found in the comparison of the results between the other groups.

#### Fat free mass

The lowest average fat free mass loss was achieved in the group of participants with the intermittent fasting and without the energy restriction, IG3 (0.2 ± 1.3 kg). The group with continuous energy restriction, IG2, achieved an average loss (0.65 ± 0.91 kg). The highest average fat free mass loss was found in the group with the intermittent fasting and energy restriction, IG1 (1.1 ± 1.0 kg). There was a statistically significant difference (IG1×IG3: p=0.027) in the fat free mass loss between the intermittent fasting with and without the energy restriction groups compared to the baseline. The comparison of the results between the other groups did not achieve any statistical significance.

A complete summary of the analysis of the outcomes in the changes in the body composition before and after the dietary intervention are presented in Table 3.

### Adverse events

The results of the incidence of adverse events in each intervention group are summarized in Table 4. No serious adverse events were reported during the intervention phase of the study. Mild adverse events were reported to varying degrees across all three groups. Statistically significant differences were achieved by comparing the frequency of fatigue between the intermittent fasting group with energy restriction, IG1 (reported in 51.9 % of participants) and the continuous energy restriction group, IG2 (reported in 22.2 % of participants), p=0.0473. The intermittent fasting group without the energy restriction, IG3, had a higher frequency of uncomfortable feeling of fullness or overeating (reported in 81 % of participants) compared to the continuous energy restriction group, IG2 (IG2×IG3: p=0.0004). Other reported adverse events are without any statistical significance between the groups.

## Discussion

The aim of the study was to assess whether intermittent fasting is as effective in reducing the body weight and body fat mass compared to continuous energy restriction. Additionally, the study aimed to investigate the effects of intermittent fasting without energy restriction on the body composition. The current evidence on the effect of intermittent fasting compared with continuous energy restriction on the body composition is not clear and provides controversial results 5,18,31,32,10,33-35.

Intermittent fasting has grown in popularity in recent years and is often recommended as a more effective option for weight loss than continuous energy restriction. This view is supported by the finding in some studies that intermittent fasting leads to a greater loss of adipose tissue 18, while helping to protect the muscle mass 31. Better maintenance of the fat free mass in response to fasting was found in the weight reduction compared with daily energy restriction 32. In fact, starvation triggers numerous hormonal adaptations, such as an increase in the serum growth hormone 36, which stimulates lipolysis, increases fat utilization and protein retention 37.

However, in our study, the intermittent fasting did not provide any advantage over the continuous energy restriction in terms of the weight loss and changes in the body composition with the comparable energy restriction. The group on continuous energy restriction without time restriction, IG2, had the highest average body fat mass loss (1.5 ± 1.2 kg). However, the observed differences compared to the group with the intermittent fasting with the energy restriction, IG1, did not reach statistical significance (p>0.99). Thus, our results are consistent with those of other studies that also showed no statistically significant difference in the weight and adipose tissue loss between intermittent fasting with energy restriction and continuous energy restriction 14,35. The duration of the fasting period may explain why the intermittent fasting group, despite having the same energy restriction as the no time restriction group, did not maintain the fat free mass as effectively. Eighteen hours is probably too short a time to allow statistically significant protective adaptive responses of the body to the fasting state. It takes about 14 hours of complete starvation to deplete the glycogen stores and begin oxidizing the fatty acids and ketone bodies. This process helps to maintain the muscle mass during weight loss. 38. Another reason why the protective adaptive response to the fasting state 32,36,37 did not show a statistically significantly greater loss of adipose tissue, as was the case in other studies, could be the presence of fatigue. Its most frequent presence was found in the participants with the intermittent fasting with energy restriction, IG1 (51.9 %; p=0.0473), which may have contributed to the reduced spontaneous physical activity.

In contrast, other studies have found that there is a greater loss of muscle mass with intermittent fasting when compared to continuous energy restriction 10,33,34. According to one of these studies 10, the results can be explained by a compensatory behavioral response that reduces the total daily energy expenditure by decreasing the spontaneous physical activity.

Another explanation for the increased loss of muscle mass during intermittent fasting could be a difference in the protein intake distribution. Some studies suggest that distributing protein consumption throughout the day has a positive influence on the muscle protein synthesis 39,40. This is due to of discovery that the anabolic effect of protein dosage is saturable 41. However, a recent study has questioned the concept of dosage and protein saturation. The study found that ingesting a single large amount of protein resulted in an increased and prolonged anabolic response, eliminating the need for another protein-rich meal in close temporal proximity 42. This may explain why intermittent fasting protocols do not appear to endanger the maintenance of muscle mass by altering the distribution of the protein intake. The results of this study support our findings that there was no statistically significant difference in the fat free mass loss between the group of participants with the intermittent fasting with energy restriction, IG1, and the group with the continuous energy restriction, IG2 (p=0.48).

The observed difference in the fat free mass loss between the IG1 and IG2 groups, which did not reach statistical significance in our study, is thus probably the result of the aforementioned compensatory behavioral response manifested by the weakening of the spontaneous physical activity. This explanation was indirectly confirmed by the increased incidence of fatigue in the group with the intermittent fasting and energy restriction IG1 (51.9%; p=0.0473). However, our study suggests that the impairment of spontaneous physical activity during intermittent fasting may not be as significant as in the study by Templeman *et al.* 10. In their study, fasting occurred for a longer period and resulted in a greater loss of muscle mass, which was statistically significant compared to the group with the comparable daily energy restriction. These results suggest that TRF may be a more sustainable long-term variant of the intermittent fasting protocol compared to alternate day fasting (ADF).

The protective hormonal adaptations to the starvation state and the anabolic response to protein dosing during intermittent fasting described above occur in parallel with the compensatory behavioral mechanisms of the organism. Therefore, in our study, the resulting effect showed comparable weight loss and similar effects on the body composition as those of the continuous energy restriction. In practice, the resulting changes in the body composition are likely to be individualized with respect to the occurrence of adverse events during the dietary intervention.

The group of participants with the intermittent fasting without energy restriction, IG3, achieved noteworthy results. Although it was the least successful in the average weight loss (1.1 ± 1.2 kg), the weight loss was achieved predominantly by reducing body fat (0.9 ± 1.1 kg). Because comparable results were not achieved by the group with the intermittent fasting with energy restriction, IG1, these results cannot be attributed only to the time window set, which probably played a major role the dietary modification, where, despite the fact that the participants were not set energy restriction, there was a significant improvement in the diet quality. All the meal plans emphasized sufficient protein, which has a higher thermic effect than other macronutrients 22,43. The body therefore uses up to 30% more energy to digest than the other macronutrients 22.

In addition, all the diet plans met the requirements for an adequate dietary fiber intake. This was achieved by a combination of a high fruit and vegetable intake and adequate consumption of fiber-rich whole grains. Therefore, the high dietary fiber content of the meal plans may have influenced not only the hunger regulation, but also the absorption of nutrients 44. It has been found 45 that the structure of plant cells in fiber-rich plant-based foods can prevent or delay the interaction between macromolecules and digestive enzymes. The cell wall, which is composed of indigestible polysaccharides, remains largely unaffected during the process of digestion. These factors can have a significant effect on the absorbability of macronutrients and thus on the actual amount of energy utilized from food. Therefore, the actual energy utilized by an isocaloric diet will differ significantly between a diet rich in dietary fiber and a diet rich in ultra processed foods 45, which are generally low in protein and dietary fiber 46. This suggestion was confirmed in another study 47, which found lower energy availability from high-fiber diets. The energy values determined by the Atwater factors were up to 11 % higher than the metabolizable energy for low-fat, high-fiber diets consisting of fruits, vegetables, and high-fiber cereals.

Another possible reason for the reduction in the adipose tissue and increased maintenance of the fat free mass in the IG3 group could be the protective response of the organism to the state of starvation 32,36,37. However, this response was not inhibited by increased fatigue, as was the case in the IG1 group.

Additionally, an important finding of our study is that the intermittent fasting combined with following the principles of a healthy diet can significantly reduce the weight and body fat mass, even without a targeted effort to induce a negative energy balance. However, the six-hour time window for food intake during weight maintenance was found to be unsuitable. It may be difficult to consume all the necessary nutrients in everyday life while following the principles of a healthy diet. In the group with intermittent fasting without energy restriction, IG3, there was a significantly higher incidence of adverse events of an uncomfortable feeling of fullness and overeating after a meal (81 %; p=0.0004). In the long term, a time window of six hours may prove to be too short, which could result in a significant risk of nutrient deficiencies.

The study findings indicate that, besides energy restriction, the success of the chosen dietary intervention is significantly influenced by the diet quality and the presence of adverse effects such as fatigue, an uncomfortable feeling of fullness after consuming large amounts of food, or hunger. In addition to successfully reducing the adiposity rates, these factors will also influence the long-term adherence to a chosen lifestyle.

### Strengths and limitations

One strength of our study was the division of participants into three intervention groups. This allowed us to compare the effect of intermittent fasting and continuous energy restriction, as well as to assess the effect of intermittent fasting in isolation from energy restriction and to compare these results with other intervention groups. Another strength of the study was the focus on the diet quality, which was planned individually for each participant. This not only allowed more accurate results, but also a high rate of adherence to the prescribed dietary regimen.

However, it is important to note that our study had certain limitations. The duration of the dietary intervention was set at 3 weeks only. The time required to prepare and consume the meals according to the diet plan was considered. The participants did not receive financial compensation for their involvement in the study and managed everything as part of their usual daily routine. Nevertheless, according to the results of a previous study 10, a period of three weeks is sufficient to detect changes in body composition.

The study included a very small number of participants, which is one of the important factors that can influence the statistical significance of the results.

The body composition was measured using BIA instead of dual energy X-ray absorptiometry (DEXA), which is considered the gold standard method. To determine the method, we relied on previous findings that DEXA results correlate well with those found by BIA 48,49.

BMR values were estimated using BIA instead of indirect calorimetry. The determination of the BMR incorporating body composition values was found to be more accurate than the BMR determination using widely used predictive equations, such as the Harris-Benedict equation 50. This is because basal energy expenditure values are strongly influenced by the body composition 51,52.

No objective method was used to quantify the physical activity of the participants. We tried to avoid any potential bias by conducting a detailed interview to obtain the most accurate data on the physical activity of the study participants.

The participants prepared their own meals based on precise meal plans. Mistakes may have been made in the preparation. We tried to eliminate this by explaining, in detail, the method of weighing the food and how to prepare it and the specific selection of the individual foods according to the food catalog.

There may also have been eating behaviors that were not reported by the participants. We tried to prevent this by regular weekly phone calls and consultations during the dietary intervention during the intervention phase of the study.

## Conclusions

The results of our study showed a comparable effect in the weight and body fat mass reduction regardless of the timing of the food intake. The appropriateness of the timing of the food intake during the dietary intervention will therefore be influenced primarily by the adherence to the chosen lifestyle, which will be significantly influenced by the presence of adverse symptoms such as fatigue, uncomfortable feelings of fullness after consuming large amounts of food or the presence of hunger. In any case, weight reduction should not only be about simply losing weight, but more importantly about achieving and maintaining good health. To achieve this, it is necessary to take in all the required nutrients in sufficient quantities and to adjust the time window for the food intake. In our study, the quality of the diet, together with the amount of energy intake, was found to be one of the most important factors influencing the body composition.

## Figures and Tables

**Table 1:** Summary of the reasons for dropping out of the intervention phase of the study

Reason	All (n=20)	IG 1 (n=7)	IG 2 (n=4)	IG 3 (n=9)
Lack of time	6	3	1	2
Acute diseases	7	3	1	3
Too large a quantity of food to eat	5	1	0	4
Failure to adhere to a defined diet (hunger, cravings, preference for foods not included in the meal plan)	2	0	2	0

**Table 2:** Characteristics of the participants at baseline

Variables	All participants (n=75)	IG 1 (n=27)	IG 2 (n=27)	IG 3 (n=21)
Sex (F/M)	52/23	15/12	20/7	17/4
Weight (kg)	83 ± 16	82 ± 16	83 ± 13	85 ± 19
Height (cm)	170.6 ± 8.9	171.6 ± 9.7	170.5 ± 8.8	169.5 ± 7.5
Age (years)	37 ± 10	37 ± 11	38 ± 10	38.1 ± 9.7
Physical activity level	1.63 ± 0.13	1.61 ± 0.12	1.65 ± 0.15	1.61 ± 0.12
Basal metabolic rate (kcal)	1580 ± 260	1610 ± 270	1540 ± 240	1570 ± 260
% Body fat	32.4 ± 8.3	29.9 ± 7.9	34.2 ± 8.7	33.4 ± 7.5
Body mass index (kg/m^2^)	28.4 ± 4.4	27.8 ± 4.3	28.4 ± 4.0	29.2 ± 5.0
(F) % Body fat	35.3 ± 7.6	34.3 ± 7.3	37.6 ± 6.7	34.2 ± 8.2
(M) % Body fat	25.8 ± 5.7	25.1 ± 5.9	24.5 ± 5.8	30.15 ± 0.90

**Table 3:** Response of the dietary interventions to the weight loss and body composition

Variable	IG 1 (IF with ER)		IG 2 (CER)		IG 3 (IF without ER)		P-value		
	Pre	∆ Post	Pre	∆ Post	Pre	∆ Post	∆IG1x∆IG2	∆IG1x∆IG3	∆IG2x∆IG3
Body mass (kg)	82 ± 16	2.3 ± 1.4	83 ± 13	2.2 ± 1.1	85 ± 19	1.1 ± 1.2	>0.99	**0.003**	**0.012**
Body mass index (kg/m^2^)	27.8 ± 4.3	0.79 ± 0.44	28.4 ± 4.0	0.76 ± 0.40	29.2 ± 5.0	0.37 ± 0.44	>0.99	**0.004**	**0.008**
Body fat mass (kg)	24.7 ± 8.7	1.3 ± 1.1	28.4 ± 9.8	1.5 ± 1.2	29 ± 11	0.9 ± 1.1	>0.99	0.73	0.17
Percent body fat (%)	29.9 ± 7.9	0.8 ± 1.1	34.2 ± 8.7	1.0 ± 1.3	33.4 ± 7.5	0.6 ± 1.2	>0.99	>0.99	0.80
Visceral fat area (cm^2^)	108 ± 42	6.8 ± 6.3	133 ± 51	9.3 ± 6.6	130 ± 52	4.6 ± 5.8	0.50	0.69	**0.043**
Waist Circumference (cm)	94.6 ± 9.9	2.7 ± 1.9	97 ± 13	2.5 ± 2.2	99 ± 15	1.7 ± 2.1	>0.99	0.31	0.56
Waist-hip ratio	0.908 ± 0.061	0.017 ± 0.023	0.933 ± 0.067	0.017 ± 0.020	0.934 ± 0.077	0.010 ± 0.024	>0.99	0.93	0.77
Soft lean mass (kg)	54 ± 12	1.01 ± 0.99	51 ± 11	0.62 ± 0.88	52 ± 11	0.2 ± 1.2	0.52	**0.032**	0.56
Fat free mass (kg)	58 ± 13	1.1 ± 1.0	54 ± 11	0.65 ± 0.91	56 ± 12	0.2 ± 1.3	0.48	**0.027**	0.54
Skeletal muscle mass (kg)	32.3 ± 7.6	0.66 ± 0.61	30.1 ± 6.8	0.37 ± 0.51	31.1 ± 7.2	0.13 ± 0.75	0.27	**0.015**	0.61
Body cell mass (kg)	37.7 ± 8.3	0.74 ± 0.67	35.3 ± 7.5	0.40 ± 0.57	36.3 ± 7.9	0.13 ± 0.83	0.52	**0.012**	0.57
Intracellular water (l)	26.3 ± 5.8	0.51 ± 0.47	24.6 ± 5.2	0.27 ± 0.40	25.4 ± 5.5	0.10 ± 0.59	0.23	**0.015**	0.69
Total body water (l)	42.1 ± 9.2	0.78 ± 0.79	39.7 ± 8.3	0.48 ± 0.70	40.8 ± 8.8	0.18 ± 0.97	0.57	**0.049**	0.67
Extracellular water (l)	15.8 ± 3.4	0.26 ± 0.35	15.1 ± 3.1	0.20 ± 0.34	15.5 ± 3.3	0.08 ± 0.40	>0.99	0.28	0.77
Bone mineral content (kg)	3.33 ± 0.71	0.059 ± 0.060	3.18 ± 0.61	0.034 ± 0.065	3.23 ± 0.68	0.001 ± 0.069	0.53	**0.010**	0.25

**Table 4:** Summary of the adverse events during the intervention

ADVERSE EVENTS	IG 1 (n=27)	IG 2 (n=27)	IG 3 (n=21)	P-value		
	No. (%) of participants	No. (%) of participants	No. (%) of participants	IG1xIG2	IG1xIG3	IG2xIG3
Fatigue	14 (51.9)	6 (22.2)	9 (42.9)	**0.0473**	0.5734	0.2089
Headache	8 (29.6)	5 (18.5)	5 (23.8)	0.5256	0.7502	0.7291
Decrease in performance	9 (33.3)	4 (14.8)	4 (19.0)	0.2021	0.3377	0.7155
Concentration disorder	6 (22.2)	2 (7.4)	1 (4.8)	0.2501	0.118	>0.99
Appetite (e.g., sweet, alcohol)	17 (63.0)	19 (70.4)	12 (57.1)	0.7734	0.7701	0.3775
Hunger	20 (74.1)	14 (51.9)	15 (71.4)	0.1581	>0.99	0.2369
Uncomfortable feeling of fullness or overeating	14 (51.9)	7 (25.9)	17 (81.0)	0.0929	0.0666	**0.0004**
Any other adverse events	5 (15.5)	3 (11.1)	2 (9.5)	0.704	0.4452	>0.99
